# Continuously Varying Critical Exponents Beyond Weak Universality

**DOI:** 10.1038/srep45004

**Published:** 2017-03-22

**Authors:** N. Khan, P. Sarkar, A. Midya, P. Mandal, P. K. Mohanty

**Affiliations:** 1 CMP Division, Saha Institute of Nuclear Physics, HBNI, 1/AF Bidhan Nagar, Kolkata 700064, India; 2 Department of Physics, Serampore College, Serampore 712201, India

## Abstract

Renormalization group theory does not restrict the form of continuous variation of critical exponents which occurs in presence of a marginal operator. However, the continuous variation of critical exponents, observed in different contexts, usually follows a weak universality scenario where some of the exponents (e.g., *β, γ, ν*) vary keeping others (e.g., *δ, η*) fixed. Here we report ferromagnetic phase transition in (Sm_1−y_Nd_y_)_0.52_Sr_0.48_MnO_3_ (0.5 ≤ *y* ≤ 1) single crystals where all three exponents *β, γ, δ* vary with Nd concentration *y.* Such a variation clearly violates both universality and weak universality hypothesis. We propose a new scaling theory that explains the present experimental results, reduces to the weak universality as a special case, and provides a generic route leading to continuous variation of critical exponents and multi-criticality.

Study of critical phenomena is based on two concepts: one is universality^1^,^2^ which states that the associated critical exponents and scaling functions are universal up to symmetries and space dimensionality, and another is scaling theory^3^ that describes the general properties of the scaling functions and relates different critical exponents. In the renormalization group approach^4^, the critical point is a fixed point governed by a unique set of relevant operators with scaling dimensions (critical exponents) which are fully independent of irrelevant operators. While a relevant perturbation may take the system to a new fixed point, the marginal one brings a possibility of continuous variation of exponents. Although the concept of universality has been verified experimentally time and again, starting from early 40s^5^ to present^6^, a continuous variation is rarely observed. A clear example is provided by Baxter^7^ who solved the eight vertex model^8^ (EVM) exactly, and Kadanoff and Wegner^9^ who provided a mapping of EVM to a two-layer Ising system with a marginal four-body interaction between the layers^10^ (similar to Ashkin Teller model^11^,^12^,^13^) that drives the continuous variation. In later years, Suzuki^14^ proposed a *weak* universality (WU) scenario where critical exponents (like *β, γ, ν* in EVM) change continuously but their ratios (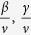 and consequently 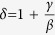) remain invariant. This WU scenario has been observed in frustrated spin systems^15^,^16^, interacting dimers^17^, magnetic hard squares^18^, Blume-Capel models^19^, reaction diffusion systems^20^, absorbing phase transitions^21^, percolation models^22^,^23^, fractal structures^24^, quantum critical points^25^, etc. The generic nature of the marginal interaction that leads to weak universality in all these different systems remains unclear.

To the best of our knowledge, most systems which show continuous variation of critical exponents obey weak universality - a few exceptions include criticality in Ising spin glass^26^, micellar solutions^27^,^28^, frustrated spin systems^29^, strong coupling QED^30^ etc. Experimentally, the continuous evolution of critical exponents with chemical substitution has been observed in URu_2−*x*_Re*x*Si_2_ (0.2 ≤ *x* ≤ 0.6) single crystals^31^. With decreasing *x*, both *γ* and *δ* decrease linearly keeping *β* fixed [(*β, γ, δ*) = (0.8, 1.0, 2.25) for *x* = 0.6 and (*β, γ, δ*) = (0.8, 0.18, 1.23) for *x* = 0.2]. By extrapolation, it has been shown that *γ* → 0 and *δ* → 1 at *x* = 0.15 at which quantum phase transition occurs. Recently, Fuch *et. al*.^32^ have observed the linear variation of exponents in the polycrystalline samples of Sr_1−*z*_Ca_*z*_RuO_3_ from (*β* ≈ 0.5, *γ* ≈ 1, *δ* ≈ 3) for *z* = 0 to (*β* ≈ 1, *γ* ≈ 0.9, *δ* ≈ 1.6) for *z* = 0.6. They have suggested that the evolution of exponents may be originating from orthorhombic distortions or additional quantum fluctuations associated with quantum phase transition at *z* = 0.7 However, whether there is a quantum critical point in Sr_1−*z*_Ca_*z*_RuO_3_ at *z* = 0.7 is still under debate^33^,^34^,^35^,^36^,^37^.

Anomalous ferromagnetic (FM) transition has also been observed in mixed valance manganites, RE_1−*x*_AE_*x*_MnO_3_ (RE: rare earth ions, AE: alkaline earth ions) either as a discontinuous transition or a continuous transition with a set of critical exponents that does not belong to any known universality or the weak universality^38^,^39^,^40^,^41^,^42^,^43^,^44^,^45^,^46^,^47^,^48^,^49^. In manganites, the nature of phases and transitions strongly depend on the bandwidth and disorder (namely quenched disorder) arising due to the size mismatch between *A*-site cations^50^,^51^. Such disorder reduces the carrier mobility and the formation energy for lattice polarons^52^, in effect *T*_*C*_ reduces, rendering the FM transition towards first-order. A system with narrow bandwidth and large disorder such as Sm_1−*x*_Sr_*x*_MnO_3_ shows a sharp first-order FM transition for *x* = 0.45 − 0.48^42^,^43^,^44^,^45^. The first-order transition is however extremely sensitive to external pressure, magnetic field, *A*-/*B*-site substitution, oxygen isotope exchange, etc. - with the application of external and internal pressure (chemical substitution) beyond a critical threshold, the transition becomes continuous^43^,^44^,^45^,^53^.

In this paper, we report two important results (i) a thermodynamic transition (*i.e.* FM phase transition in (Sm_1−*y*_Nd_y_)_0.52_Sr_0.48_MnO_3_) where critical exponents *β, γ, δ* vary continuously, and (ii) a new scaling theory that explains the present experimental results and reduces to the weak universality as a special case. We propose that, to obey the scaling relations consistently, the variation of critical exponents are constrained to have specific forms. This scaling hypothesis naturally leads to two special cases which have been realized earlier, namely the weak universality^14^ (where *δ* is fixed) and the strong coupling QED^30^ (fixed *γ*). A more generic scenario is the one which allows simultaneous variation of *all* the critical exponents in an intrigue way, leading to a multi-critical point where the phase transition becomes discontinuous. This scenario is verified experimentally in a comprehensive and systematic study of FM phase transition in (Sm_1−*y*_Nd_*y*_)_0.52_Sr_0.48_MnO_3_ single crystal. For higher doping concentration *y* > 0.4 the FM transition is found to be continuous, but to our surprise, the critical exponents exhibit continuous variation with Nd concentration *y*, starting from (*β, γ, δ*) = (0.16, 1.27, 9.30) at *y* = 0.5 to (0.36, 1.38, 4.72) at *y* = 1. Within error limits, *y* = 1 belongs to the universality class of Heisenberg model in three dimension (HM3d). The proposed scaling hypothesis successfully explains the continuous variation of exponents in the present system, and predicts that the transition is discontinuous for doping *y* ≲ 0.37 which has been observed experimentally^43^,^44^.

## Results and Discussion

### Critical temperature and exponents

**Figure 1:**
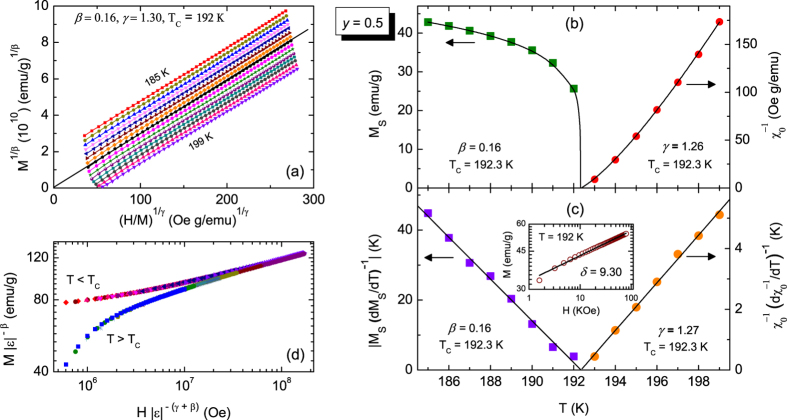
(**a**) Modified Arrott plot [*M*^1/*β*^ vs 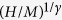] isotherms (185 K ≤ T ≤ 199 K in 1 K interval) of (Sm_1−*y*_Nd_y_)_0.52_Sr_0.48_MnO_3_ (*y* = 0.5) single crystal. Solid lines are the high-field linear fit to the isotherms. The isotherm (at *T* = 192 K) closest to the Curie temperature (*T*_*C*_ = 192.3 K) almost passes through the origin in this plot. (**b**) Temperature dependence of spontaneous magnetization, *M*_*S*_ (square) and inverse initial susceptibility, 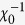 (circle). Solid lines are the best-fit curves. (**c**) Kouvel-Fisher plots of *M*_*S*_ and 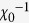. Inset shows log scale plot of M(H) isotherm at *T* = *T*_*C*_ (**d**) Scaling collapse of *M* − *H* curves following Eq. (2), indicating two universal curves below and above *T*_*C*_.

Let us set notations by reminding that in absence of magnetic field (*H*) the spontaneous magnetization of the system vanishes as *M*_*S*_(0, *ε*) ~ (−*ε*)^*β*^ and the initial susceptibility diverges as 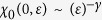 as the critical point is approached, *i.e.,* when (*T/T*_*C*_ − 1) ≡ *ε* → 0. Again at *T* *=* *T*_*C*_, the magnetization varies as *M(H, T*_*C*_) ~ *H*^1/δ^^54^. To estimate the critical exponents *β, γ* and *δ*, we need to know *T*_*C*_ accurately. To do so, we exploit the linearity in Arrott-Noakes equation of state^55^


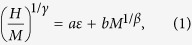


where *a, b* are non-universal constants. The correct choice of *β* and *γ* can make the isotherms of *M*^1*/β*^ versus (*H/M*)^1/γ^ a set of parallel straight lines with *one* unique critical isotherm that passes through the origin. This is explained in Fig. 1(a) for *y* = 0.5 and the self consistency is achieved for the values *β* = 0.16, *γ* = 1.30. The isotherm *T* = 192 K passes almost through the origin. From the intercepts of these parallel straight lines on *M*^1*/β*^ and (*H/M*)^1/γ^ axes, we obtain *M*_*S*_ and 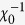 for different temperatures which are shown in Fig. 1(b). The best power-law fit gives *β* = 0.16, *γ* = 1.26 and *T*_*C*_ = 192.3 K. These estimates are in fact consistent with Kouvel-Fisher criteria^56^ which predict that in the scaling regime, both 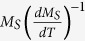 and 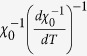 are proportional to *T* with proportionality constants *β*^−1^ and *γ*^−1^ respectively; which is shown in Fig. 1(c). Another critical exponent *δ* is found from *M*-*H* isotherm at 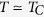 (here *T* = 192 K). The log-scale plot of *M* vs. *H*, as shown in the inset of Fig. 1(c), is linear with slope *δ* = 9.3 Following the same method, we have determined the critical exponents and *T*_*C*_ s’ for *y* = 0.6, 0.8 and 1.0 (see section I of Supplementary information for details) which are listed in Table 1 along with the critical exponents for HM3d. In the present system, the variation of *T*_*C*_ with *y* can be explained in terms of bandwidth and *A*-site cation size disorder^51^ of the system. With increasing *y*, the bandwidth increases whereas disorder decreases; both of these effects enhance *T*_*C*_ (see section II of Supplementary information for details). It is clear from the Table 1 that the exponents for *y* = 1 are very close to that of HM3d, whereas they deviate substantially and vary systematically when *y* is decreased. Clearly, *β* decreases by *two*-fold whereas *δ* increases almost by the same amount as one goes from *y* = 1 to *y* = 0.5 At the same time, the Widom scaling relation *β* + *γ* = *βδ* is satisfied for each *y* = 0.5, 0.6, 0.8, 1. This indicates that the change in *γ* has to be minimal, as observed here (see section IV of Supplementary information for details). The variation of critical exponents with respect to a system parameter contradicts universality, moreover it violates WU as *δ* varies along with *β* and *γ*. The focus of current work is to address this issue in details, but first let us ask whether the scaling hypothesis, crucial for the description of near critical systems, is valid here.

**Figure 2:**
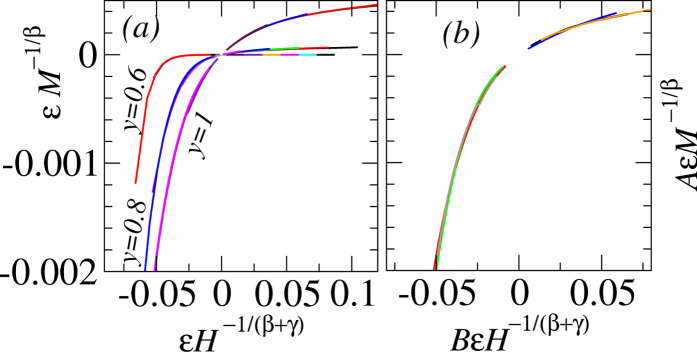
(**a**) Scaling collapse of *M* − *H* curves for *y* = 0.6, 0.8 and 1 following Eq. (3). Although they appear different, the scaling functions for *y* = 0.5, 0.6, 0.8, 1 can be collapsed onto each other (shown in (**b**)) by rescaling of axis. The values of (*A, B*) are (1080, 2.8), (7.2, 1.6) and (1, 1) for *y* = 0.6, 0.8 and 1, respectively.

For a thermodynamic system near FM transition, magnetization *M* depends on *H* and *ε* and follows a universal scaling form^54^


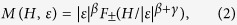


where *F*_+_ is for *T* > *T*_*C*_ and *F*_*−*_ is for *T* *<* *T*_*C*_. The utility of universal scaling function lies in the fact that the *M* − *H* curves obtained for different *T* (near *T*_*C*_) can be collapsed onto a single curve when one plots 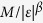 versus 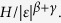 This scaling collapse is shown in Fig. 1(d) for *y* = 0.5 (and in section I of Supplementary information, for other values of *y*); the two branches in this curve correspond to super- and sub-critical phases. For *y* = 0.6, 0.8 and 1.0, the data collapse is also shown in Fig. 2(a), where we use an alternative but equivalent form of Eq. (2),


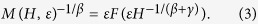


It is advantageous to use this form as the two branches of Eq. (2) are now merged to a single function *F* with its argument 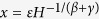 extending from the sub-critical (*x* < 0) to the super-critical (*x* *>* 0) regimes. A very good quality data collapse confirms that the scaling hypothesis is in place and the estimated value of *T*_*C*_ and critical exponents obtained through several prescriptions are unambiguous and self-consistent.

### The scaling hypothesis

In the following, we propose a new scaling ansatz which explains the experimental findings presented here. Since diverging fluctuations are known to be the origin of power-laws (see ref. 57 for a proof for non-equilibrium systems, which also holds trivially for equilibrium), we start with a scaling relation that relates the exponents of energy and magnetic fluctuations (i.e., *α* and *γ*) with that of diverging correlation length associated with criticality:


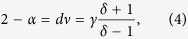


where *ν* and *α* are exponents associated with correlation length 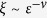 and specific heat 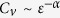, respectively and *d* is the dimension. The first relation relates the diverging correlation length to the energy fluctuation whereas the second relation relates the same to the magnetic fluctuation (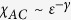 and 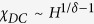). If a set of exponents originates from *one* underlying universality class then they must relate to the exponents of the parent universality (*α*_0_, *ν*_0_, *γ*_0_, *δ*_0_) as


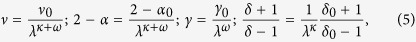


so that the universal scaling relation [Eq. (4)] remains valid. Here, *λ* is the marginal parameter that drives the continuous variation and *ω* and *κ* are parameters yet to be determined. In Eq. (5), the variation of *δ* and *γ* are considered to be independent and equivalently one may vary *η* and *ν* independently and deduce the variation of other exponents from scaling relations (see section III of Supplementary information for details).

Clearly *κ* = 0 = *ω* is the parent universality class having unscaled (*λ* = 1) exponents. Other special cases are when *κ* or *ω* vanishes. When *κ* = 0, the continuous variation is governed by *c* = *λ*^*ω*^, In this case, we may set *ω* = 1 without loss of generality and identify *c* ≡ *λ* as the physical parameter that drives a continuous variation


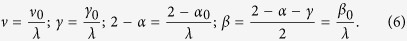


This scenario is already known as weak universality where 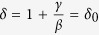 and 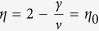 are universal as in the old universality argument^14^. Another special case is *ω* = 0 and *κ* is set to be unity. Here *γ* remains invariant and


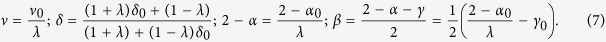


This variation, with *λ* being the gauge coupling constant, has been observed in strong coupling QED^30^. Now we turn our attention to the generic case where *κ* > 0 and *ω* is set to unity (generality is not compromised); the consequent variation of critical exponents is given by


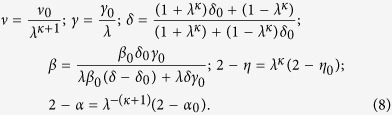


This generic variation of exponents satisfies Widom scaling relation *δ* = 1 + *γ/β* for any *λ*, provided the parent universality class also obeys the same.

We further include a possibility that this generic variation may lead to *β* → 0, a special limit where the phase transition becomes discontinuous. At this multi-critical limit, the usual scaling theory demands *γ* → 1 and *δ*^−1^ → 0^58^. The first requirement can be met if the multicritical point occurs at *λ* = *γ*_0_ and the second requirement determines


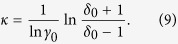


**Figure 3:**
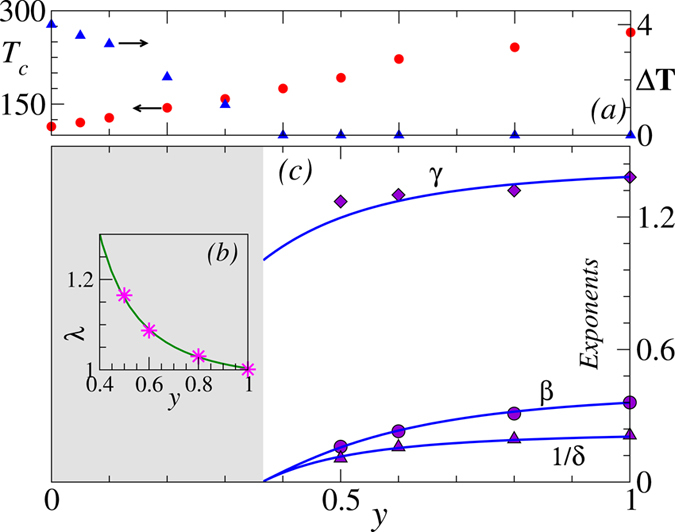
(**a**) Critical temperature *T*_*C*_ for heating cycle (red circle) and the thermal hysteresis width Δ*T* (blue triangle) for different Nd concentration *y*. The values for *y* < 0.5 are taken from ref. 44. (**b**) The proposed scaling hypothesis has one free parameter *λ* that maps to *y* -the best choice gives 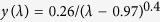 (solid line). (**c**) Critical exponents for different *y* from experiments (symbol) are compared with Eq. (8) (solid lines). Since 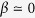 at 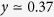, one expects a discontinuous transition for *y* ≲ 0.37 (also observed in (**a**)).

For the present experimental system, the parent class is HM3d (at *y* = 1) and accordingly *κ* = 1.29 (from Eq. (9) and Table 1). At doping *y* = 1, we may set *λ* = 1, but a general correspondence between *y* and the marginal parameter *λ* can not be obtained unless we know the correct interaction Hamiltonian. The best choice of *λ* that matches the exponents for *y* = 0.5, 0.6, 0.8 and 1 turns out to be 1.165, 1.087, 1.03 and 1.001 respectively. The best fit between *λ* and *y* with 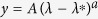 yields *A* = 0.26, λ^*^ = 0.97 and *a* = −0.4, which is shown in Fig. 3(b). This functional form 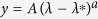 is used further in Eq. (8) to get continuous variation of *β, γ* and 1/*δ* with respect to *y* (shown as solid line in Fig. 3(c)). Moreover Eq. (8) suggests that the transition becomes discontinuous if *λ* > *γ*_0_ = 1.386 which corresponds to *y* ~ 0.37. In fact, a very sharp growth in the ordered moment just below *T*_*C*_ along with thermal hysteresis (shown in Fig. 3(a)) has been observed in the present system for *y* < 0.4^44^. In a related system (Sm_1−*y*_Nd_*y*_)_0.55_Sr_0.45_MnO_3_, where Sr concentration differs slightly, the FM transition remains first order for 0 ≤ *y* ≤ 0.33^43^.

To emphasize that the new scaling hypothesis is indeed in work, we further investigate the scaling functions. If the critical behavior for different *y* is related to HM3d, one expects that the seemingly different universal scaling functions *F* in Fig. 2(a) must collapse onto each other if *x* and *y* axes are scaled by suitable constants. This is described in Fig. 2(b) - a good collapse of scaling functions for *y* = 0.5, 0.6, 0.8,1 to a unique universal form further supports that the observed criticality is only a rescaled form of Heisenberg fixed point.

## Conclusions

In conclusion, we have made a comprehensive study of critical phenomenon in (Sm_1−*y*_Nd_*y*_)_0.52_Sr_0.48_MnO_3_ single crystals with 0.5 ≤ *y* ≤ 1. The values of critical exponents (*β, γ, δ*) measured for *y* = 1 are consistent with Heisenberg universality class in three-dimension, whereas the same for *y* = 0.5, 0.6, 0.8 are far from any known universality class. All these exponents vary continuously with *y*, but they seem to obey the standard scaling laws following a single equation of state. The variation of exponents is not new to critical phenomena as it can be generated by a marginal interaction, but most examples (though there are a few exceptions) in both theoretical and experimental studies satisfy the weak universality^14^ where (*β, γ, ν*) vary but (*η, δ*) are fixed. We argue that, to be consistent with scaling, the continuous variation must occur in specific ways. Two special cases are, (a) *δ* remains unchanged, which leads to the weak universality, and (b) *γ* is unaltered, which results in a kind of variation observed in strong coupling QED^30^. This generic universality scenario, which leads to a multi-critical point, explains the continuous variation of critical exponents observed in (Sm_1−*y*_Nd_*y*_)_0.52_Sr_0.48_MnO_3_ and correctly predicts the possibility of a discontinuous transition for doping *y* ≲ 0.37.

A marginal interaction that could provide variation of critical exponents beyond weak universality remains elusive. In particular, it is not clear, how or why a marginal operator is generated in all the above experimental conditions to drive continuous variations in specific ways. It is certainly challenging to devise a microscopic theory to accommodate this phenomenon.

## Experimental Methods

The single crystals of (Sm_1−*y*_Nd_*y*_)_0.52_Sr_0.48_MnO_3_ with *y* = 0.5, 0.6, 0.8 and 1.0 were prepared by floating zone technique under oxygen atmosphere^59^,^60^. Single crystallinity was confirmed by the Laue diffraction. The dc magnetization measurements were performed using a Quantum Design magnetic property measurement system (MPMS SQUID VSM) in fields up to 7 T. The data were collected after stabilizing the temperature for about 30 minutes. External magnetic field was applied along the longest sample direction and data were corrected for the demagnetization effect.

## Additional Information

**How to cite this article:** Khan, N. *et al*. Continuously Varying Critical Exponents Beyond Weak Universality. *Sci. Rep.* **7**, 45004; doi: 10.1038/srep45004 (2017).

**Publisher's note:** Springer Nature remains neutral with regard to jurisdictional claims in published maps and institutional affiliations.

## Supplementary Material

Supplementary Information

## Figures and Tables

**Table 1:** Critical exponents of (Sm_1−*y*_Nd_*y*_)_0.52_Sr_0.48_MnO_3_.

*y*	*T*_*C*_ (K)	*β*	*γ*	*δ*
0.5	192.3 ± 0.3	0.16 ± 0.01	1.27 ± 0.03	9.30 ± 0.2
0.6	222.5 ± 0.3	0.23 ± 0.01	1.30 ± 0.02	6.31 ± 0.1
0.8	241.3 ± 0.2	0.31 ± 0.01	1.32 ± 0.01	5.14 ± 0.03
1.0	265.3 ± 0.2	0.36 ± 0.01	1.38 ± 0.01	4.72 ± 0.01
HM3d	−	0.365	1.386	4.82

Error bars are derived from the least squares fitting analysis.
