# Supplementary material for: Continuously Varying Critical Exponents Beyond Weak Universality
Source: Sci Rep. 2017 Mar 22;7:45004. doi: 10.1038/srep45004 (PMC5361157; doi:10.1038/srep45004)
Supplement: Supplementary Information [file srep45004-s1.pdf]

Supplementary Information : Continuously Varying Critical Exponents Beyond Weak Universality

N. Khan[1], P. Sarkar[2], A. Midya[1] , P. Mandal[1] and P. K. Mohanty[1,*]

[1] *CMP Division, Saha Institute of Nuclear Physics, HBNI, 1/AF Bidhan Nagar, Kolkata 700064, India*

[2] *Department of Physics, Serampore College, Serampore 712201, India*

[*] *Correspondence and requests for materials should be addressed to P.K.M. (email: pk@saha.ac.in)*

In this supplement, we provide additional details of the experiments and the scaling theory.

I. CRITICAL BEHAVIOR OF $(Sm_{1-y}Nd_y)_{0.52}Sr_{0.48}MnO_3$

Figure I.1 describes determination of the critical point and exponents of $(\mathrm{Sm}_{1-y}\mathrm{Nd}_y)_{0.52}\mathrm{Sr}_{0.48}\mathrm{MnO}_3$ for $y = 1.0$. Figure I.2 describes the same, but for different doping $y = 0.6$ and 0.8.

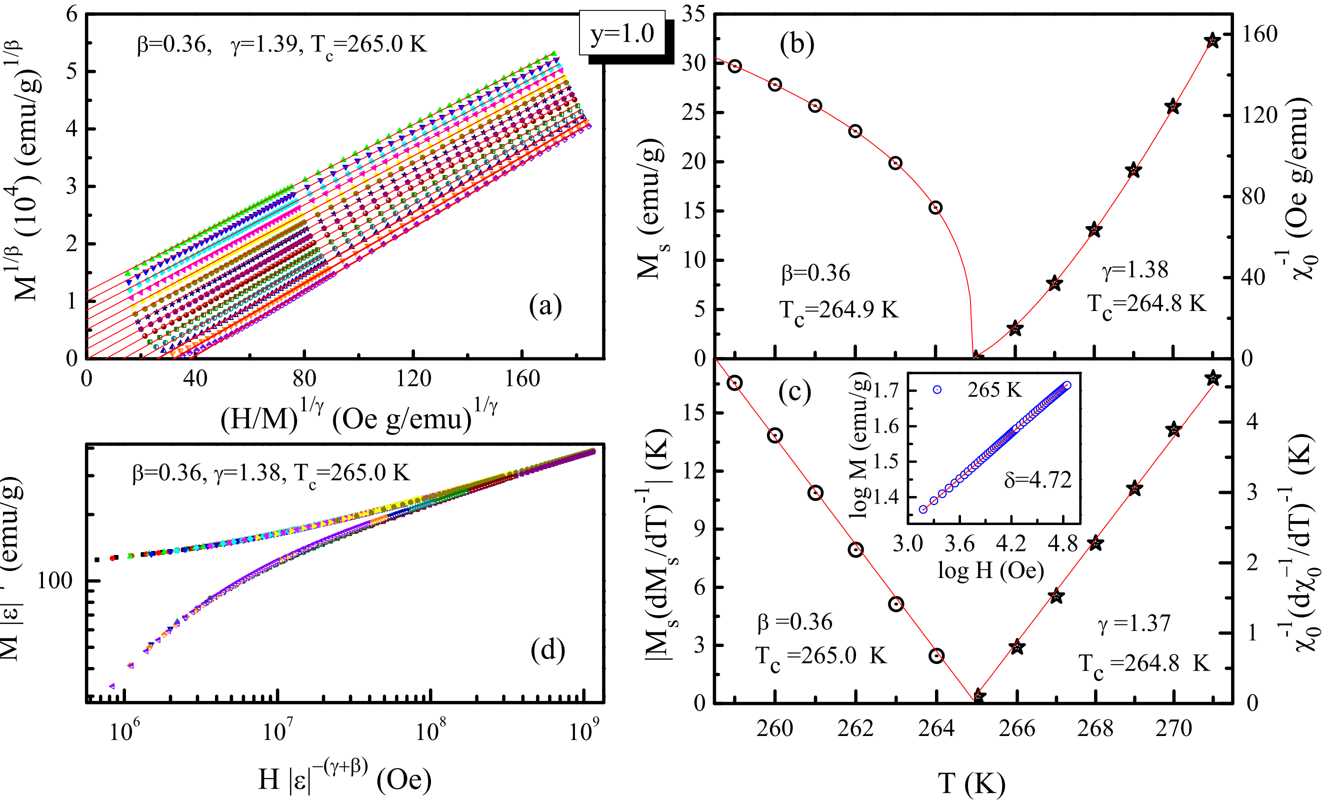

FIG. I.1: (a) Modified Arrott plot $[M^{1/\beta}$ vs $(H/M)^{1/\gamma}]$ isotherms (258 K $\leq T \leq$ 271 K in 1 K interval) of $(\mathrm{Sm}_{1-y}\mathrm{Nd}_y)_{0.52}\mathrm{Sr}_{0.48}\mathrm{MnO}_3$ (y=1) single crystal. Solid lines are the high-field linear fit to the isotherms. The isotherm at 265.0 K almost passes through the origin in this plot. (b) Temperature dependence of spontaneous magnetization, M_S (circle) and inverse initial susceptibility, χ^{-1} (star). Solid lines are the best-fit curves. (c) Kouvel-Fisher plots of M_S and $\chi_0{}^{-1}$. Inset shows log-log plot of M(H) isotherm at $T = T_C$ (d) Scaling collapse of $M - H$ curves: $M|\epsilon|^{-\beta}$ is an universal function of $H|\epsilon|^{-(\beta+\gamma)}$, indicating two universal curves below and above T_C.

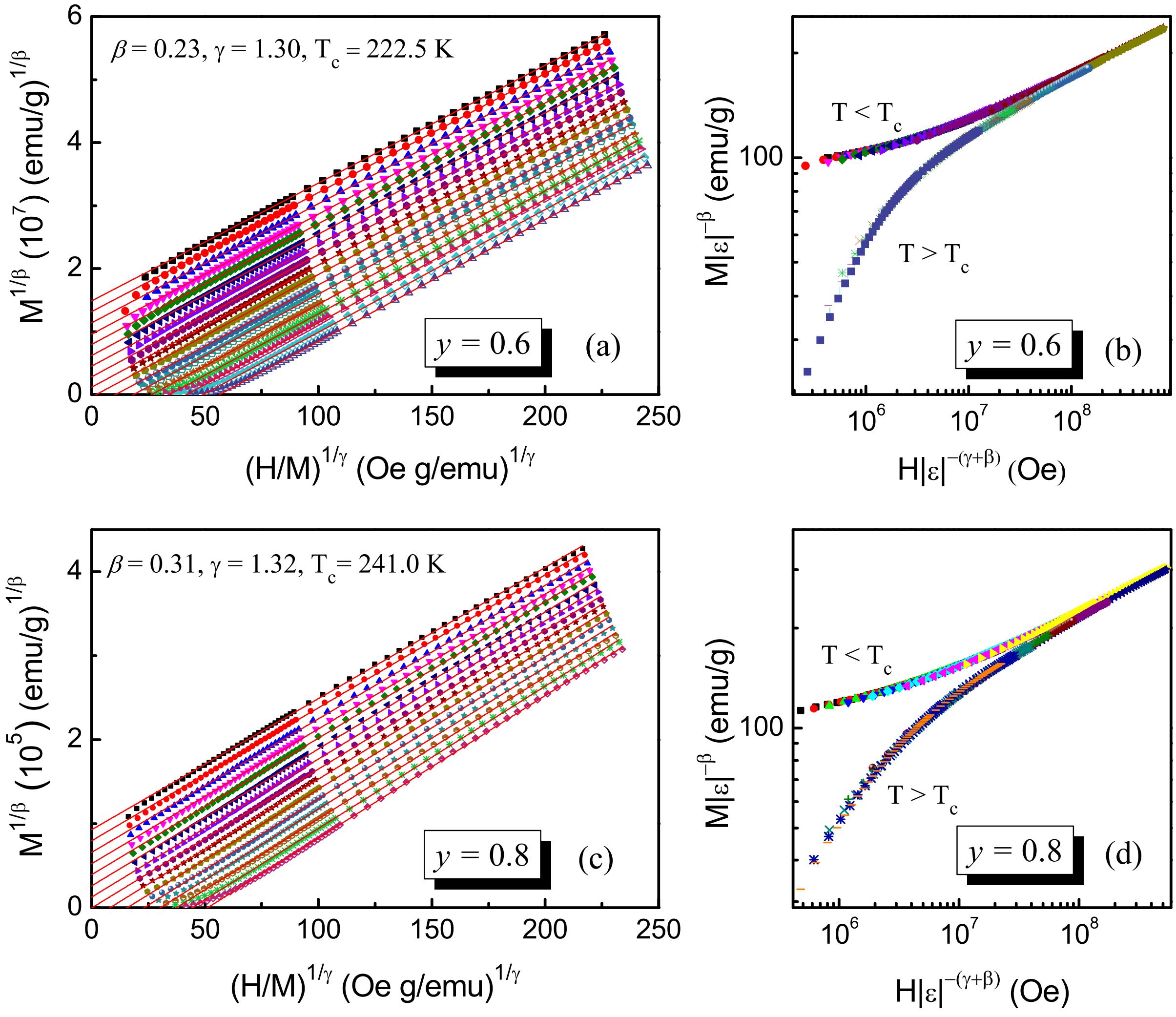

FIG. I.2: (a) Modified Arrott plot $[M^{1/\beta}$ vs $(H/M)^{1/\gamma}]$ for (a) $y = 0.6$ and (c) 0.8. The critical isotherm, which almost passes through the origin gives an estimate of respective $T_C \simeq 222.5$ K and 241 K. (b) and (d) : A plot of $M|\epsilon|^{-\beta}$ against $H\epsilon|^{-(\beta+\gamma)}$ show scaling collapse for $y = 0.6, 0.8$ respectively.

II. VARIATION OF T_C WITH Nd CONCENTRATION

For mixed valance manganites, $RE_{1-x}AE_xMnO_3$ (RE: rare-earth ion and AE: alkaline-earth ion), one of the key parameters to determine the nature of phase is the transfer interaction of the e_g-state conduction electron between the neighboring Mn sites or the effective one-electron band-width of e_g-band. The bandwidth of the system can be controlled by tuning average A-site cation radius which is given by

$$\langle r_A \rangle = \sum_i x_i r_i,$$

where x_i and r_i are the atomic fraction and ionic radii of i-type ions at A-site, respectively. Other than bandwidth, A-site cation size disorder (also known as quenched disorder), arises mainly from the size difference in ionic radii of A-site cations, also plays an important role in determining the nature of phases and phase transitions. The magnitude

of quenched disorder is usually quantified by the variance in the ionic radii of the A-site cations,

$$\sigma^2 =< r_A^2 > - < r_A >^2 = \sum_i x_i r_i^2 - \left(\sum_i x_i r_i\right)^2 .$$

The effect of $\langle r_A \rangle$ and σ^2 on the ferromagnetic-metal to paramagnetic-insulator transition temperature of $RE_{0.7}AE_{0.3}MnO_3$ perovskites by using various RE (La, Pr, Nd, Sm) and AE (Ca, Sr, Ba) ions has been extensively studied [1, 2] and their results show that T_C can be expressed as

$$T_C(\langle r_A \rangle, \sigma^2) = T_C(r_A^0, 0) - p_2(r_A^0 - \langle r_A \rangle)^2 - p_1\sigma^2, \tag{II.1}$$

where r_A^0 is the ideal radius for an undistorted cubic perovskite and $T_C(r_A^0, 0)$ is an estimate of the transition temperature for an ideal ($\langle r_A \rangle = r_A^0$), disorder-free ($\sigma^2$=0) system. We have calculated $\langle r_A \rangle$ and σ^2 of

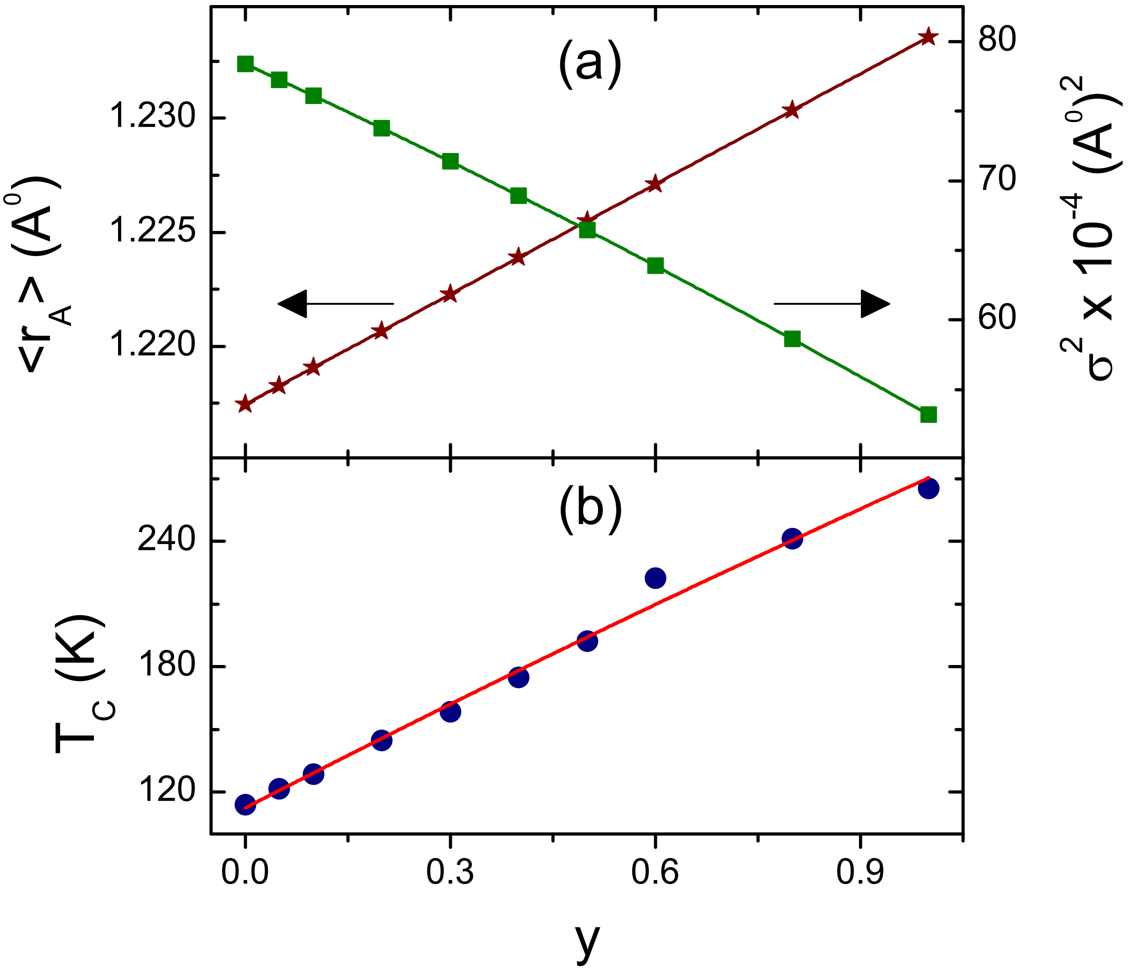

FIG. II.3: (a) $\langle r_A \rangle$ and σ^2 of $(Sm_{1-y}Nd_y)_{0.52}Sr_{0.48}MnO_3$ as a function of y. (b) T_C vs. y phase diagram. Solid circle: experimental data points. Red line: best fit curve according to Eq. (II.2).

$(Sm_{1-y}Nd_y)_{0.52}Sr_{0.48}MnO_3$ for different values of y by using standard ionic radii (ninefold coordination) with values 1.132, 1.163, and 1.31 $\mathring{A}$ for Sm^{3+}, Nd^{3+}, and Sr^{2+}, respectively. With increasing y, $\langle r_A \rangle$ increases while σ^2 decreases [as shown in Fig. II.3 (a)]. As both $\langle r_A \rangle$ and σ^2 depend on y, T_C can be expressed as a function of y as

$$T_C(y) = T_C(r_A^0, 0) - p_2[r_A^0 - \langle r_A \rangle(y)]^2 - p_1[\sigma^2(y)]. \tag{II.2}$$

Following Eq. (II.2) we try to fit the experimentally measured T_C for different y; the best fit (Fig. II.3 (b)) is obtained for $T_C(r_A^0, 0)$=530 ± 10 K, p_2=61700 ± 1200 K/$\AA^2$ and p_1=19752 ± 1000 K/$\AA^2$. The variation of σ^2 with y is not linear and hence T_C is not linear with y.

III. AN ALTERNATIVE SCALING

In the main text we have chosen the exponents γ and δ to scale independently

$$\gamma \to \frac{\gamma}{\lambda^\omega}; \frac{\delta+1}{\delta-1} \to \frac{1}{\lambda^\kappa}\frac{\delta+1}{\delta-1}. \tag{III.3}$$

But, since magnetic phase transitions are associated with *two* independent critical exponents, one can vary any two independently and fix variation of others through scaling relations. In theoretical studies, η and ν are natural choices as they can be obtained from the two-point correlation function,

$$\langle S(\vec{R})S(\vec{R}+\vec{r})\rangle - \langle S(\vec{R})\rangle^2 = \frac{e^{-|\vec{r}|/\xi}}{|\vec{r}|^{d-2+\eta}}, \tag{III.4}$$

where the correlation length $\xi \sim (T_c - T)^{-\nu}$. Also, η satisfy another hyper scaling relation,

$$2-\eta = d\frac{\delta-1}{\delta+1} = \frac{\gamma}{\nu}. \tag{III.5}$$

Thus the variation we propose in the main text (Eq. (5)), is equivalent to

$$2-\eta = \lambda^\kappa(2-\eta_0) \; ; \; \nu = \frac{\nu_0}{\lambda^{\kappa+\omega}}. \tag{III.6}$$

Other exponents can be derived as they are functions of (η, ν),

$$\gamma = \nu(2-\eta) \; ; \; \alpha = 2 - d\nu \; ; \; \beta = \frac{\nu}{2}(d-2+\eta) \; ; \; \delta = \frac{d+2-\eta}{d-2+\eta}$$

when scaling relations hold.

IV. SMALL VARIATION OF THE SUSCEPTIBILITY EXPONENT γ

In $(Sm_{1-y}Nd_y)_{0.52}Sr_{0.48}MnO_3$ we have studied the critical behaviour of the ferromagnetic transition for 0.5≤ y ≤1.0. For $y = 1$, the critical exponents β, γ and δ are consistent with 3D Heisenberg universality class. As y decreases, all three exponents vary continuously : the variation of β and δ are as large as two fold in comparison to $y = 1$, whereas the variation of γ with y is only about 8% ($\gamma = 1.38$ for $y = 1.0$ and $\gamma = 1.27$ for $y = 0.5$). Such a small variation may provoke one to think that γ remains unaltered with y. However this can not be true for the following reasons. First, if γ remains invariant then according to our proposed scaling theory, the variation of β and δ should be according to Eq. (7) of the main manuscript. In Fig. (IV.4) we have shown this variation in dotted line taking $\gamma = \gamma_0 = 1.386$. The solid lines in this figure represent the variation according to Eq. (8), where all three exponents β, γ and δ vary continuously. Clearly, the experimentally observed values of β, γ, δ fits well with Eq. (8), as compared to Eq. (7) where γ remains constant.

Secondly, in the present system there is a multicritical point at $y \approx 0.4$ (experimentally observed earlier [3, 4]) below which continuous ferromagnetic transition becomes first-order, where γ has to be 1 (along with $\beta \to 0$ and $\delta \to \infty$ [5]). Thus, with decreasing y from 1.0, γ should decrease systematically from 1.38 (the value of γ for y=1.0) and should approach to 1 for $y \approx 0.4$. Note, that the change in γ is at most 27%. This is due to the fact that Widom scaling $\gamma = \beta(\delta - 1)$, being respected here, neutralizes the large increase of δ with corresponding decrease in β.

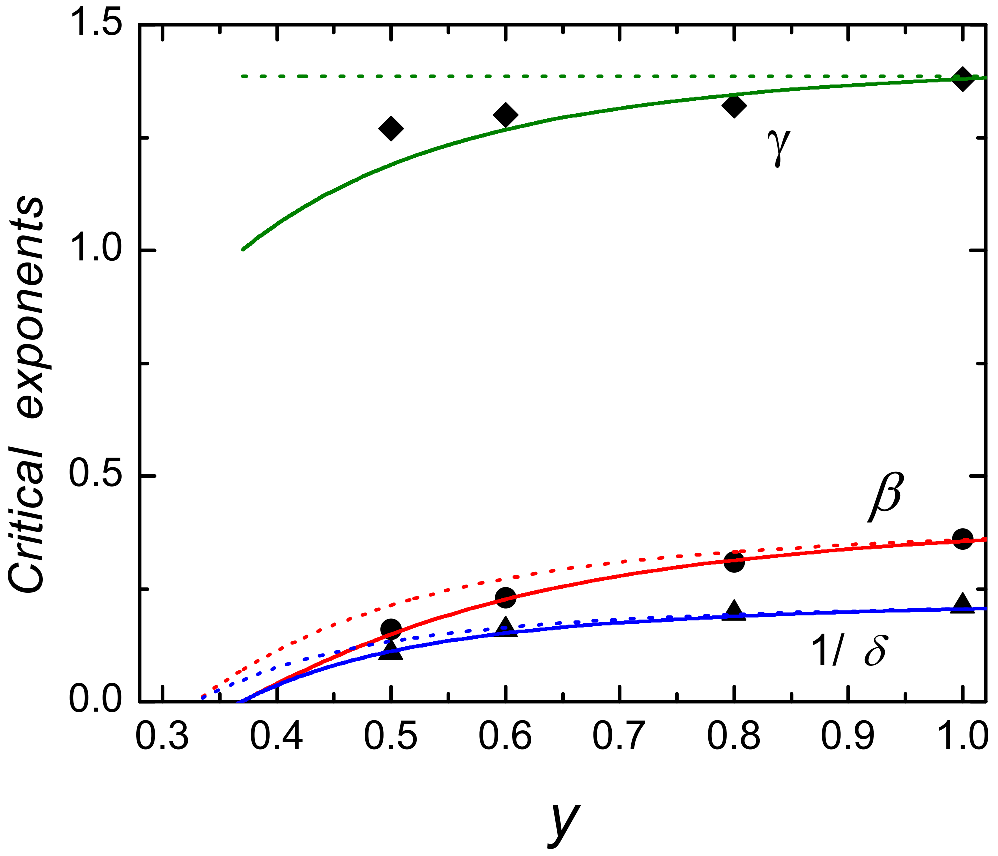

FIG. IV.4: Critical exponents β, γ, and δ of $(Sm_{1-y}Nd_y)_{0.52}Sr_{0.48}MnO_3$ for different y ($0.5\leq y \leq 1.0$). Solid symbols represent experimentally determined critical exponents. Dotted and solid lines are the variation of exponents according to Eq. (7) and (8) of the main manuscript, respectively.

References

[1] Rodriguez-Martinez, L. M. & Attfield, J. P. Cation disorder and size effects in magnetoresistive manganese oxide perovskites. *Phys. Rev. B* **54**, R15622 (1996).

[2] Rodriguez-Martinez, L. M. & Attfield, J. P. Disorder-induced orbital ordering in $L_{0.7}M_{0.3}MnO_3$ perovskites.*Phys. Rev. B* **63**, 024424 (2000).

[3] Demkó, L. *et al.* Multicritical end point of the first-order ferromagnetic transition in colossal magnetoresistive manganites. *Phys. Rev. Lett.* **101**, 037206 (2008).

[4] Sarkar, P. *et al.* Role of external and internal perturbations on the ferromagnetic phase transition in $Sm_{0.52}Sr_{0.48}MnO_3$. *Phys. Rev. B* **79**, 144431 (2009).

[5] Fisher, M. E. & Berker, A. N. Scaling for first-order phase transitions in thermodynamic and finite systems. *Phys. Rev. B* **26**, 2507-2513 (1982).
